# Amplified warming in tropical and subtropical cities under 2 °C climate change

**DOI:** 10.1073/pnas.2502873123

**Published:** 2026-02-03

**Authors:** S. Berk, M. M. Joshi, C. M. Goodess, P. Nowack

**Affiliations:** ^a^Climatic Research Unit, School of Environmental Sciences, University of East Anglia, Norwich, Norfolk NR4 7TJ, United Kingdom; ^b^Institute of Theoretical Informatics, Institute of Meteorology and Climate Research Atmospheric Trace Gases and Remote Sensing, Karlsruhe Institute of Technology, Karlsruhe 76131, Germany

**Keywords:** urban heat island, climate change, machine learning, urban climate

## Abstract

Cities are often warmer than rural surroundings due to a phenomenon known as the urban heat island, which can be influenced by various factors, such as regional climate and land surface types. Under climate change, cities face not only the challenge of increasing temperatures in their surrounding hinterland but also the challenge of potential changes in their heat islands. However, even high-resolution global Earth system models (ESMs) with “urban tiles” can only properly resolve the largest urban areas or megacities. Here, we address these limitations by applying a process-based statistical learning model to ESM outputs to provide projections of changes in land surface temperature (LST) for 104 medium-sized cities of population 300 K to 1 M in the subtropics and tropics. Under a 2 °C global warming scenario, annual mean LST in 81% of these cities is projected to increase faster than the surrounding area. In 16% of these cities, mostly in India and China, mean LST is projected to increase by an additional 50-112% above ESM projections of the surrounding area. Our findings underscore the importance of investigating the specific effects of climate change on urban heat exposure.

## Significance

Urban heat stress under climate change is an increasing concern, as most cities are already warmer than their rural surroundings, heightening their vulnerability to rising temperatures and exposing a large share of the global population. While Global Climate Models are essential for projecting future temperature changes, their relatively coarse scale limits their ability to capture the trends of smaller cities. To bridge this gap, projected changes in land surface temperature in medium-sized cities are created and compared to surrounding regions, identifying areas where the urban warming rate is faster than rural surroundings. Our analysis shows low-resolution projections likely underestimate future urban warming in most cities, highlighting the need for deeper study.

The urban heat island (UHI) is a phenomenon whereby the temperature in a city differs from the surrounding rural area, typically being warmer. This leads to increased heat-related health risks for urban inhabitants in comparison to their rural counterparts (1). In 2018, it was estimated that over half the world’s population resided in cities and this proportion is projected to increase to 68% by 2050 (2). Climate change results in rising global temperatures and increased frequency of extreme heat events (3), which can have severe human health impacts including increased mortality (4–6).

UHIs are influenced by both climate and city attributes (e.g., city area, rural aridity, and landcover) (7), all of which can change over time. A deeper understanding of climate change related shifts in UHI intensities will inform city planners as they design cities aiming to optimize human comfort and health, and enable evidence based adaptation planning. However, modeling and projecting changes in UHI on a global scale remains a challenge. ESM or Global Climate Model (GCM) outputs have spatial resolutions larger than the scale of most cities due to limitations in computational power. As urban landcover only represents a small fraction of the earth’s surface, its inclusion is not essential for projecting mean global temperature changes and is often not represented, although studies utilizing more complex urban schemes exist (8). Even in such cases however, ESMs can have limitations in their ability to simulate the UHI: for instance, ESMs with tiled schemes for predicting surface temperature separately for urban and surrounding rural areas have urban and rural air temperatures that are identical (i.e., the same model grid point) (9). Regional climate models have higher resolutions and provide detailed understandings of the urban microclimate, and downscaling of GCMs is an active research field (10). However, such models are also constrained by computational expense. This limits their ability to model many cities simultaneously at a high enough resolution to capture a medium-sized city (11).

The above limitations mean that projections of the impacts of climate change on the UHI are also limited to either the largest cities (12), or to smaller cities in certain geographical regions, at a lower resolution (13). Indeed, much of the current research focus of the UHI is on megacities, which represent just 12% of the urban population (14, 15). Furthermore, several regions of the world are underrepresented in the UHI literature, e.g., Africa and South and Central America (15, 16). Typically, as cities expand the intensity of their UHI also grows (17). However, it is observed that saturation of the UHI with city size occurs in very large cities (e.g., London) (18–20). Cities where saturation of the UHI has occurred may respond differently to climate change than those medium-sized cities where this point has not yet been reached. A complete picture of UHI behavior under climate change can therefore only be gained by addition of the examination of such medium-sized cities.

A barrier to making observation-based global studies of the UHI is that weather station data are irregular and sparse in coverage, and air temperature sensor networks in cities are rare and have limited temporal coverage (21). Furthermore, the methodologies and placements of such sensor networks are inconsistent, making city to city comparisons based on air temperature networks problematic (22). Satellite data, on the other hand, have a resolution of 1 km and global coverage. In addition, a positive correlation exists between urban LST and air temperature (23, 24) because LST controls air temperature in the lower layers of the atmosphere, although spatially within a city, cool and hot spots do not necessarily overlap (24). In this paper, we use Earth system model (ESM) projections and a process-based machine learning (ML) model of the surface UHI (SUHI), the UHI based on LST differences, to make projections of total surface temperature change in selected medium-sized cities in the tropics and subtropics under 2 °C global warming [a critical benchmark established at the 2015 Paris agreement (25)].

Statistical or ML approaches have been used before in SUHI studies to explore relationships between predictor and target variables (26), but have not been employed to predict SUHI changes in the future. Our process-based model is based on predictors that are known to affect climate on city-sized scales, e.g., precipitation, humidity, vegetation (*Methods*). The selected ML model (Regression Enhanced Random Forest) is chosen for its capacity to perform well under extrapolation circumstances (27), which is necessary for studies of future global change; this property is confirmed via various test-train splits (*Methods*). Our approach is complementary to ESM-based projections of the SUHI, and detailed studies into the microclimate of individual cities would enhance the findings of this study, in particular for those highlighted as most impacted by climatic changes.

## Results

### A Statistical Learning Approach for SUHI Projections.

Here, we present our results for the day-time SUHI at 13:30 h. The observed values of the past SUHIs (*SI Appendix*, Fig. S1) and results for night-time (*SI Appendix*, Fig. S2), when changes in SUHI with climate change are generally much smaller, can be found in the supplementary material. First, an overview of the selected cities is given. Next, we describe our ML model and evaluate its performance. We then describe the projections made by combining the ML model with ESM outputs.

**Fig. 1.**
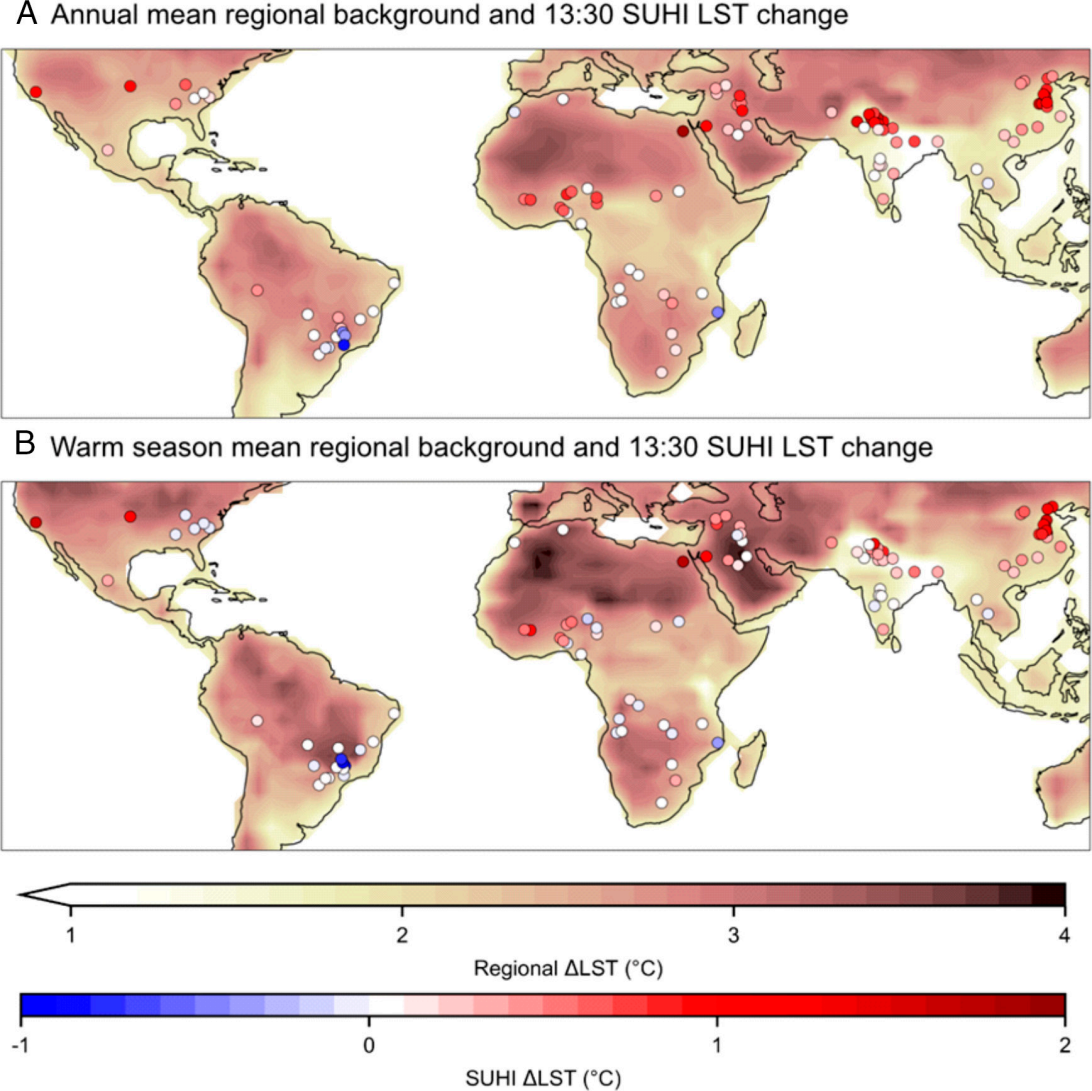
Locations of selected cities and projected LST changes for the background regional area and the additional SUHI-driven changes with 2 °C warming. Maps show regional changes in mean LST projected by the ESMs, with the additional LST changes in the city projected by the ML model for (*A*) annual values and (*B*) the warm season.

From a dataset of global urban areas (2), we impose city selection criteria to return a subset containing medium-sized cities with additional restrictions to remove nonclimatic influences. For example, coastal cities or those in mountainous regions are not included. Selection criteria are listed in *Methods* section. The locations of the 104 selected cities are shown in Fig. 1.

**Fig. 2.**
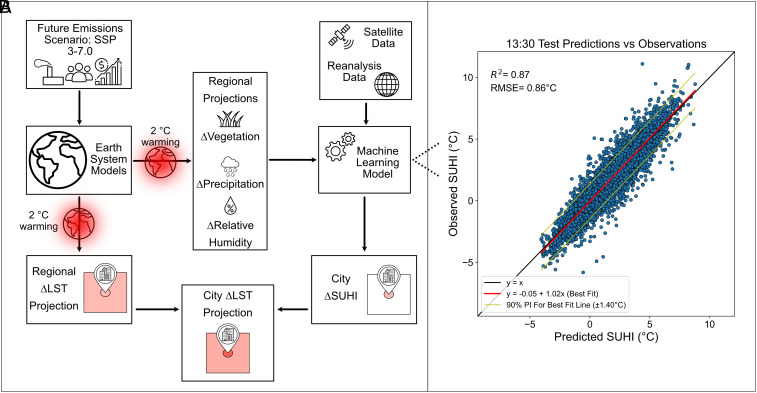
Modeling overview and ML model performance. (*A*) Schematic showing the process to generate projections of changes in SUHI and regional and city LSTs. (*B*) Scatterplot of ML SUHI predictions (horizontal axis) versus observations (vertical axis) for the test data. Data were split by alternate years, with test data odd years. Each point represents the monthly mean SUHI observation and prediction for all 104 cities.

Fig. 2*A* outlines the procedure used to generate the changes in urban LSTs. The ML model, Regression Enhanced Random Forest (RERF) (27), is set up to predict SUHI magnitudes from factors such as urban–rural vegetation differences and relative humidity. These factors are acquired from satellite and reanalysis data for the model development. Changes in these drivers under a global 2 °C warming, obtained from CMIP6 ESM projections, are then used to project changes in the SUHI (*Methods*).

Our ML model performs well for present-day climates across the selected cities, successfully predicting SUHI magnitudes for a range of observed values, as shown in Fig. 2*B*. Across all cities, the test data have overall performance statistics of R-squared 0.87 and RMSE 0.86 °C, giving confidence in the ability of the model to make projections of the SUHI on unseen data. We consider various validation scenarios to ensure a robust model for the required application (*Methods*).

### SUHIs Increase With 2 °C Warming.

**Fig. 3.**
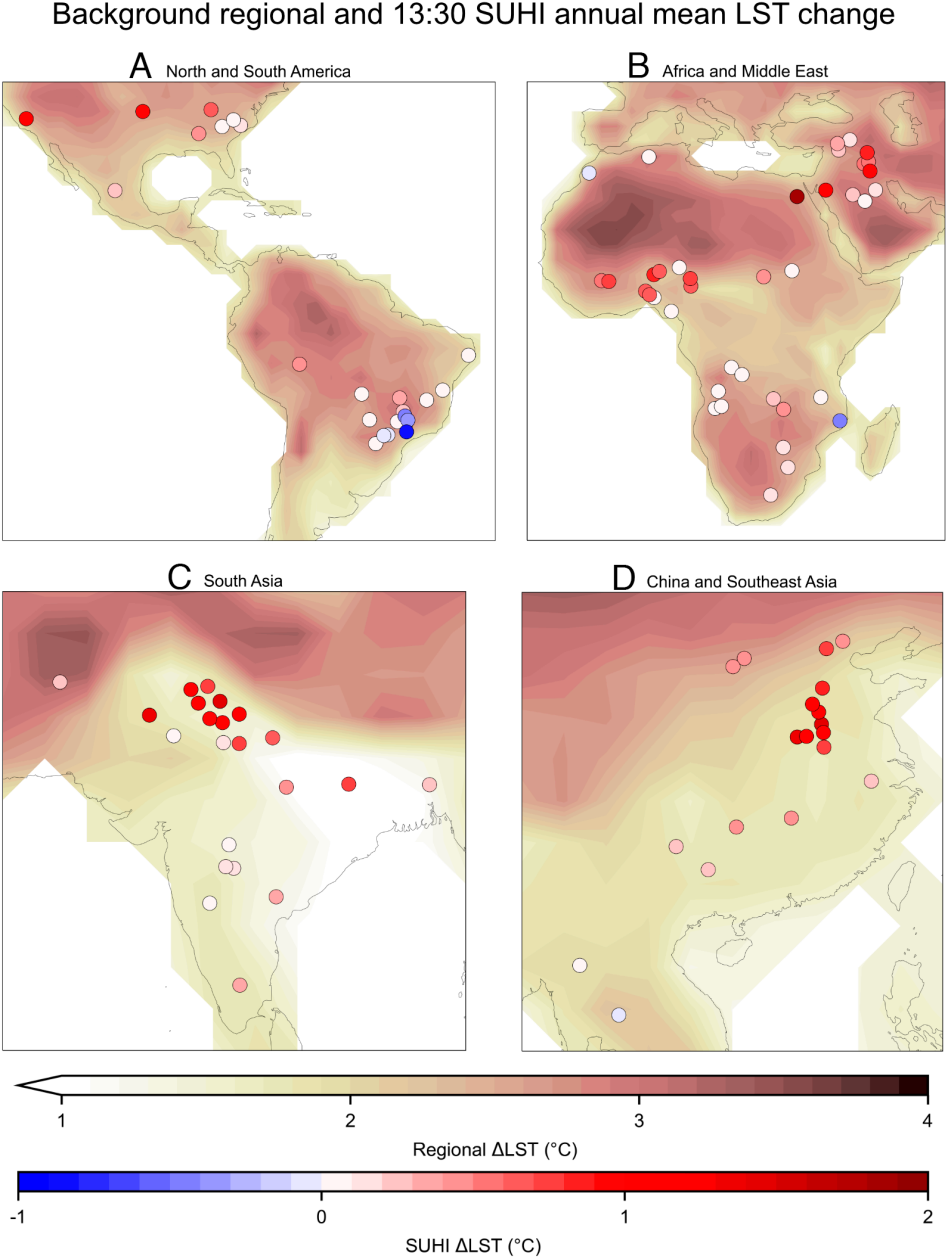
A closer view of the projected annual LST changes for the background regional area and the additional SUHI-driven changes. The map in Fig. 1*A* is split into regions showing (*A*) North and South America, (*B*) Africa and Middle East, (*C*) South Asia, and (*D*) China and Southeast Asia. The same figure for the warm season can be seen in *SI Appendix,* Fig. S3.

For most of the 104 cities, the current SUHI is projected to become more positive. This is apparent in Fig. 1. Under 2 °C warming, 81% of 13:30 SUHIs are projected to increase in their annual mean. This change is in addition to the regional background warming projected by ESMs also shown in Fig. 1. The overall mean of this amplification is 0.4 °C, increasing the overall change in city temperature (urban ∆LST) from 2.2 °C (the ESM regional ∆LST) to 2.6 °C. Cities in the Middle East, India, and China all undergo large additional annual warming, as shown in Fig. 1*A*. Fig. 3 zooms in on the annual changes shown in Fig. 1. In the Middle East increases in SUHI are a particular cause for concern as these regions are already very hot, and also face a considerable increase in ESM-based regional LST. In these areas, the current SUHI is negative (an urban cool island), due to greater vegetation and irrigation in the urban area in contrast to the rural (28). The projected increase in the urban LST in these regions indicates the SUHI becoming less negative, and in some cases, positive.

Increases in SUHI magnitude are especially likely to impact human health during the warmest months of the year (29). To investigate this, the data were split into four calendar quarters and the warmest season defined as that with the highest mean 2 m air temperature for each region. This warm season projected change can be seen in Fig. 1*B* (with a magnified version in *SI Appendix*, Fig. S3) which shows that 75% of the SUHIs increase, with an overall mean change of 0.3 °C (with ESM ∆LST being 2.4 °C and urban ∆LST 2.7 °C). Warm season increases in SUHI magnitude are particularly noticeable for cities in Northeastern China.

### Major Shifts in Highly Populated Regions.

For many highly populated countries, such as India and China, projected changes in the SUHI are shown to be particularly pronounced in comparison to background levels of warming (Fig. 3). For all the studied cities in India, mean LST is projected to increase by an additional 45% above ESM projections of the surrounding area, and in China by an additional 40%. A major reason for this is the influence of vegetation, which is associated with increased cooling due to evapotranspiration and water retention (30). Predictor importances, determined using accumulated local effects (ALE) (31), find vegetation to be a strong influencer on the SUHI magnitude (*SI Appendix*, Fig. S4). ESM projections of large-scale changes to vegetation or moisture availability, which have a cooling influence on rural areas, do not typically affect cities to the same extent, as they are made up of artificial impervious surfaces and drainage systems that carry away surface water (32). In the areas where there are increases in regional vegetation (ESM projections can be seen in *SI Appendix*, Fig. S5), the SUHI becomes more positive. Here, these changes in vegetation, which lead to an increased magnitude in urban–rural vegetation difference, are responsible for the largest changes in the SUHI. In parts of Brazil the opposite effect is seen (Fig. 3), and the SUHI becomes smaller.

**Fig. 4.**
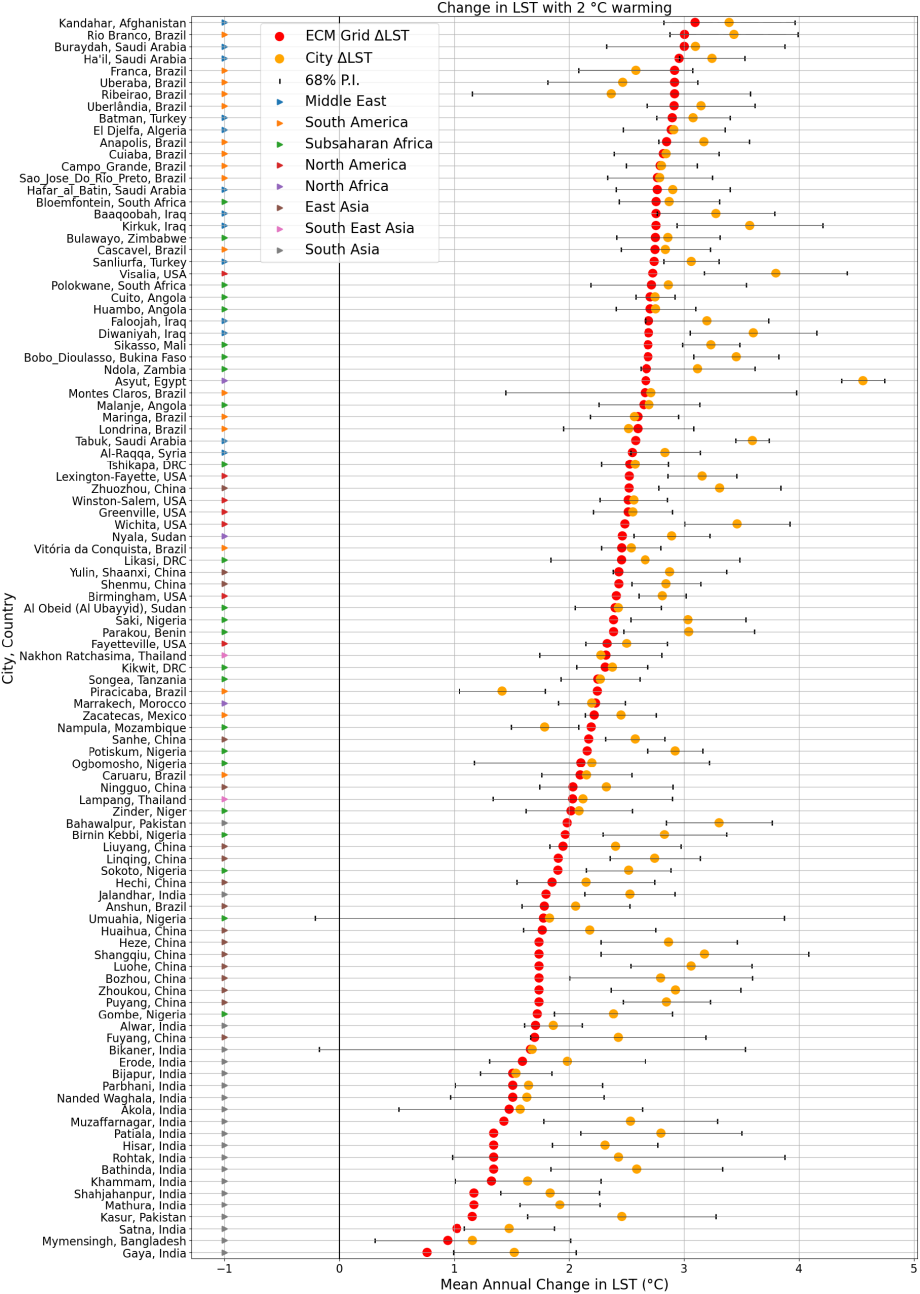
Projected LST changes with and without SUHI changes under 2 °C warming for the 104 individual cities. The red dots show the median projected LST changes for the overall ESM grid cell region. Orange dots show the median ML projected changes in the city LST additional to this, driven by the changes in the ESM climate variables. A 68% prediction interval for the ML model is shown in black, representing the likelihood that the true value of each projection lies within this range.

Fig. 4 summarizes how the inclusion of city-specific projections can have a substantial influence on the overall ∆LST as a function of ESM ∆LST. While only 3 city regions experience an increase above 3 °C based on ESM LST, 26 cities experience increases in median urban modeled LST above 3 °C. For two of the cities, Patiala, India, and Kasur, Pakistan, the additional change in SUHI results in the city ∆LST being twice that of the ESM projection.

Prediction intervals, based on the ML model, are shown in Fig. 4. The cities which have small projected changes in SUHI tend to have the largest prediction intervals, indicating these SUHIs are less influenced by the input climatic variables in the ML model.

When changes in SUHI are considered on top of the changes in regional LST, it is clear that almost all of the cities studied undergo larger LST increases than their rural hinterlands. The overall influence of including city-specific projections, rather than simply examining the ESM grid cell, skews the probability distribution of ∆LST toward larger magnitudes for both the annual and warm season mean values (*SI Appendix*, Fig. S6).

## Discussion

We have investigated the effects of climate change on the daytime SUHI of 104 medium-sized cities in the tropics and subtropics, which are currently home to over 50 million inhabitants. City temperatures are already amplified due to the UHI in most regions, with exception of the most arid (33, 34), and globally all areas face increases in temperature due to climate change (35). On top of these known factors, we have demonstrated the potential for urban warming to be amplified in many cities, i.e., city LSTs increasing faster than ESM projections suggest. We note that such a trend has already been observed over the last 20 y (36).

Our results are of immediate relevance to policymakers who will need to account for the increased hazards many urban citizens will face over the coming decades. The cities studied here are located in the warmer parts of the world, which makes this increase even more impactful for human health and the urban environment (37, 38). More generally, medium-sized cities represent a large proportion of global cities with more than 2.5 times as many cities in this category than cities with over 1 million population (2). Our method, which combines state-of-the-art climate change projections with process-based ML models, enables more informed planning for these future risks, and aims to complement the current body of research using physical modeling approaches.

The projected SUHI increases are particularly noticeable in the highly populated regions of north India and northeastern China. This is concerning as both these areas are projected to experience more frequent and intense heatwaves (39, 40). In hot temperatures, outdoor workers are subject to numerous negative impacts of heat exposure (41) and economic impacts should they forgo a day’s work. India is projected to require large cooling demands in the future, which is problematic as the infrastructure may not be able to cope with this increased load, and the costs are prohibitive for many (42). Increased energy usage also brings consequences for climate change mitigation. The need for UHI mitigation and heat adaption in these regions is therefore even more pressing.

A caveat of this study is that city expansion has not been considered. Including future urban expansion would likely lead to an even greater warming compared to the above projected changes (43). Relaxing the city selection criteria to include more cities would make the results more generalizable, although the addition of variables into the ML model would increase complexity. In particular, including coastal cities and those near large waterbodies, where humidity is more important, and an important aspect of human health and comfort, is a source for future research.

## Methods

### City Selection.

The included cities had a population between 300,000 and one million and a latitude of less than 40°. Additionally, the surrounding features (lakes, hills, oceans) of cities were considered in order to control other variables and isolate the impact of climate (this could be relaxed in future work). These nonclimatic influences are removed to ensure the model is capturing relationships based on physical processes related to climate, rather than other differences between selected cities.

An overview of the criteria and datasets used is provided below.

• Population: 300,000 to 1,000,000 (2)
• Location: <40 ° Latitude (2)
• City distance: >42 km from any other city with >300,000 population (2)
• Coastal distance: >100 km from shoreline (44)
• Water proximity: >50 km from lakes >50 km wide or >22 km from lakes >1 km wide (45)
• Topography: <±150 m (SD) in elevation within 55 km^2^ of surrounding area (46)
• City area: >5 km^2^ city area in 2002 (47)

### Generating the SUHI and Predictor Variables.

The dataset used to develop the ML model was generated using satellite and reanalysis data. The workflow diagram for the model build and use can be seen in Fig. 2*A*. Here, we explain the processing for the satellite and reanalysis data used to quantify the SUHI and generate input variables for the ML model.

For satellite data, cloud contamination thresholds were set so at least 70% of the overall (rural + urban) area and 50% of the urban have usable pixels. For images deemed acceptable, any remaining poor-quality pixels (predefined in the datasets via quality flags) were masked in the analysis to promote accuracy.

SUHI was quantified as the difference between the monthly mean city LST and the surrounding rural reference area;

[1] $$SUHI=\frac{1}{n_{\mathrm{urban}}}\sum_{i}^{n_{\mathrm{urban}}}{\mathrm{LST}}_{\mathrm{urban}}-\frac{1}{n_{\mathrm{rural}}}\sum_{i}^{n_{\mathrm{rural}}}{\mathrm{LST}}_{\mathrm{rural}},$$

where *LST_urban_*, *n_urban_*, *LST_rural_*, and *n_rural_* represent the LST and number of urban and rural pixels, respectively.

The rural reference area was defined as a rectangular box surrounding the city, where the city takes up the 10% of the area in the center. The city and all other urban pixels in the area are masked out. The satellite data utilized for SUHI quantification are outlined below. The temporal span of the data was determined as the overlapping period for all data, from 2002 to 2020. Spatially, landcover data, used to flag urban versus rural LST pixels, was regridded to the same grid as the LST data (1 km).

• Terra LST 8-D Global (MOD11A2) (48)
• ESA Land Cover Climate Change Initiative: Global Land Cover Maps, Version 2.0.7 (47, 49)

A number of SUHI quantification methods in two categories were considered. The first category, chosen in this study, is to assess the mean of urban pixel LST. The second category involves an assessment of the “peak” SUHI (50), aiming to understand the LST differential where the city is the warmest. Methods in category 1, involving mean LSTs, tended to show highly correlated results, with similar magnitudes and distributions (*SI Appendix*, Fig. S7). The additional method investigated was using the same urban area, but the rural reference was set as a 5 km buffer from the city bounds.

The second category, investigating the warmest LST pixels within a city is less correlated with the mean methods. Additionally, when the ML models are fit with these methods as target variable, performance decreases, indicative of drivers of peak SUHI that are nonclimatic and may be more related to urban features such as building density.

The predictor variables used (selected based on maximizing R-squared) are monthly means of the following.

• Relative humidity (RH)
• Total precipitation (TP)
• Urban enhanced vegetation index (EVI_U)
• Urban–rural enhanced vegetation index difference (EVI_D)
• Log_10_ of city area (LOG_AREA)
• Urban–rural white sky albedo difference (WSA_D)
• Urban–rural elevation difference (ELEVATION_D)
• SD of urban elevation (STD_ELEVATION_U)

Climate variables (RH and TP) were taken to be the mean of the entire area inspected for the analysis, including both the city and the rural area. For satellite-derived variables, which have resolution of 1 km or less, urban and rural values are determined by calculating the mean result for the areas marked urban and rural in the ESA landcover data.

To ensure the ML model is process based, these variables were chosen as they are known to influence the SUHI (30, 33, 51, 52), and relate to heat and moisture exchange in the surface energy balance. The variable relationships with the predictions made are tested through model evaluation techniques such as ALE (31) (*SI Appendix*, Fig. S4) to ensure the ML model accurately reflects the physical processes that cause the SUHI to vary. To reduce multicollinearity, all predictor variable candidates were checked for correlations, and any pair with a Pearson’s correlated coefficient of |r| > 0.7 (53) are not considered in the model together.

City area is calculated using the ESA landcover data (47) and elevation variables using the topography (46). The additional satellite and reanalysis datasets used are as follows.

• RH, TP from ERA-5 (54)
• EVI_U, EVI_D from MODIS MYD13A2, MOD13A2 (55, 56)
• WSA_D from MODIS MCD43A3, MCD43A2 (57, 58)

The urban–rural difference variables were derived using the same rural and urban areas, as well as Eq. **1** applied in SUHI quantification. After quality control, 22,177 points representing monthly mean values were retained for training and testing the ML model. The smallest cities based on 2020 area are Bahawalpur (Pakistan) and Mymensingh (Bangladesh) and the largest Birmingham (USA). Taking the annual mean for each city, overall, there are more positive SUHIs (76%) than negative (*SI Appendix*, Fig. S1). For positive SUHIs, the mean was 2.2 ±1.5 °C (±SD). Negative SUHIs had a mean of −1.1 ± 0.9 °C. Exploratory analysis revealed the predictor most strongly correlated with the SUHI (based on Pearson’s correlation coefficient) was EVI_D, showing a strong negative correlation (r = −0.77). For most cities, annual mean EVI_R exceeded EVI_U, aside from 4 cities in arid areas. The greatest rural EVI is in Cascavel (Brazil) in the DJF (southern hemisphere summer) months at 0.56 and the lowest in Ha’il (Saudi Arabia) which persists at a mean of 0.06 y-round. A correlation matrix of all predictor variables and SUHI is provided in *SI Appendix*, Fig. S8. Not all predictor variables showed linear relationships with SUHI. For example, LOG_AREA was found to be positively correlated (r = 0.39) with SUHI magnitude in cities exhibiting positive SUHIs, consistent with previous findings (59,60, 61), but negatively correlated (r = −0.24) with negative SUHIs.

### Developing the ML Model.

The chosen ML model, RERF (27), combines the strengths of Ridge Regression (RR) and Random Forest Regression (RFR) in a two stage hybrid setup. In the first stage, a “base” model (RR in this case) is trained on the input features and target variable. This step captures the more linear components of the relationship between the predictor and the response, providing a stable parametric fit. This model then generates preliminary predictions on the training set, which are subtracted from the observed target values to produce residuals. In the second stage, these residuals are modeled using RFR. This nonparametric method captures more complex, nonlinear interactions and dependencies the RR cannot account for. The final RERF prediction is then obtained by summing the RR baseline and RFR prediction of the residuals. The model therefore capitalizes on the benefits of both methods. Fitting a linear and parametric relationship between variables via the Ridge base model, which is more robust in a changing climate system than other ML techniques (62), and the flexibility of a nonparametric Random Forest to model more intricate, nonlinear patterns. The RERF has been shown to perform well under extrapolation circumstances (27), where a standalone RFR would typically degrade in performance due to its reliance on training domain similarity.

Data were split into test and training data and RERF hyperparameters were tuned using fivefold cross-validation on the training data only. The split was done based on odd and even years, but validation was also undertaken using various splits (e.g., early and later years) and performance remained similar. Additionally, two specific extrapolation tests were done. One using the warmest 20 cities as the test dataset and the remaining 84 cities as training, and a second using the negative or smallest 10% plus largest 10% of SUHI magnitudes as the test data and remaining 80% of data as the training. Details of these tests can be seen in *SI Appendix*, Fig. S9. Comparisons with RFR and RR are also shown, to demonstrate the improved ability of the RERF to extrapolate, where RFR cannot. Additionally, including input variables from a wide variety of climate regimes results in the range of the training data more likely to encompass potential future climate scenarios.

Before using the RERF to make projections, it was refitted on the entire dataset using the hyperparameters from cross-validation. This gives the best constrained model on the largest possible training data range, still with the objectively best hyperparameter settings for the REFR fit.

### Merging the ML Model with ESM Projections.

Changes in the future SUHI are investigated by combining the RERF functions learned from observations with climate model projections for future regional changes (i.e., areas surrounding the cities considered) for the predictor variables from the most recent phase of the Coupled Model Intercomparison Project (CMIP)—CMIP6. ESM projections are used to quantify potential future changes in vegetation and climate, so they can then be added into the dataset of predictor variables.

A key challenge is that ESM climate projections show different rates of warming due to the forcings, feedbacks, and parameterizations used (63). An alternative approach is to analyze climate at a 2 °C global mean temperature rise from preindustrial (64). This is additionally relevant to policymakers as it is easier for those without expertise in climate modeling to understand. Here, we use the SSP3-7.0 pathway, the medium to high emissions scenario (65).

To use ESM projections in the RERF, variables are converted according to the following preprocessing steps:

1. Calculate a preindustrial mean global temperature for each ESM, defined as the mean global temperature from 01/01/1850 to 01/01/1900.
2. Find the 20-y period where the mean global temperature is 2 °C higher than preindustrial baseline. This will be known as future period.
3. Regrid the ESMs so they are all on the same grid. This is the coarsest grid, CanESM5, which has spatial resolution 2.8° latitude x 2.8° longitude.
4. Use the ESM outputs from the preindustrial period to get a baseline for the climate (surface RH and TP) and vegetation (LAI) variable outputs.
5. Use the ESM outputs from the future period (2 °C global mean warming from preindustrial) to get a projection for the future climate and vegetation variables.
6. Calculate the change in the climate and vegetation variables using these two ESM outputs. This gives a change in LAI, RH, and TP for each ESM (5 total). By looking at the difference between preindustrial and 2 °C warming in the model rather than the absolute prediction for each predictor variable, some bias adjustment is implicitly performed on the ESMs.
7. The changes in the predictor variables are then added to observations. This means the resolution of the variables will remain that of the observations, and not those of the ESMs. This technique is known as the delta change method (66).

An implicit assumption of the approach is that climate forcing will be constant throughout an ESM grid box.

Well studied and validated ESMs, with the required variables and scenario available from CMIP6 were chosen. The ESMs are as follows: CanESM5 (67–69), CNRM-CM6-1 (70–72), ACCESS-ESM1-5 (73–75), IPSL-CM6A-LR (76–78), and UKESM1-1-LL (79–81).

### Statistical Analysis.

Prediction intervals for the REFR model were calculated using the SE of the model performance for each individual city. The 68% prediction interval corresponds to plus and minus one SE. ALE plots were used to assess how the individual variables contribute to the overall outcome of a model, and ensure these relationships agree with known physical processes. ALE plots depicting variable influences on prediction outcomes can be seen in *SI Appendix*, Fig. S4. Pearson’s correlation coefficient (r) (82) was used to quantify the strength and direction of the linear relationship between variables.

## Supplementary Material

Appendix 01 (PDF)

## Data Availability

Code is publically available on Zenodo (83). All data used in this study are publically available, including city population and location (2), coastal distance (44), water proximity (45), topography (46), landcover (47), LST (48), vegetation index (55, 56), albedo (57, 58) and CMIP6 ESM (68, 69, 71, 72, 74, 75, 77, 78, 80, 81). All other data are included in the manuscript and/or *SI Appendix*.
