# Supplementary material for: Amplified warming in tropical and subtropical cities under 2 °C climate change
Source: Proc Natl Acad Sci U S A. 2026 Feb 3;123(6):e2502873123. doi: 10.1073/pnas.2502873123 (PMC12890902; doi:10.1073/pnas.2502873123)
Supplement: Supplementary file 1 — Appendix 01 (PDF) [file pnas.2502873123.sapp.pdf]

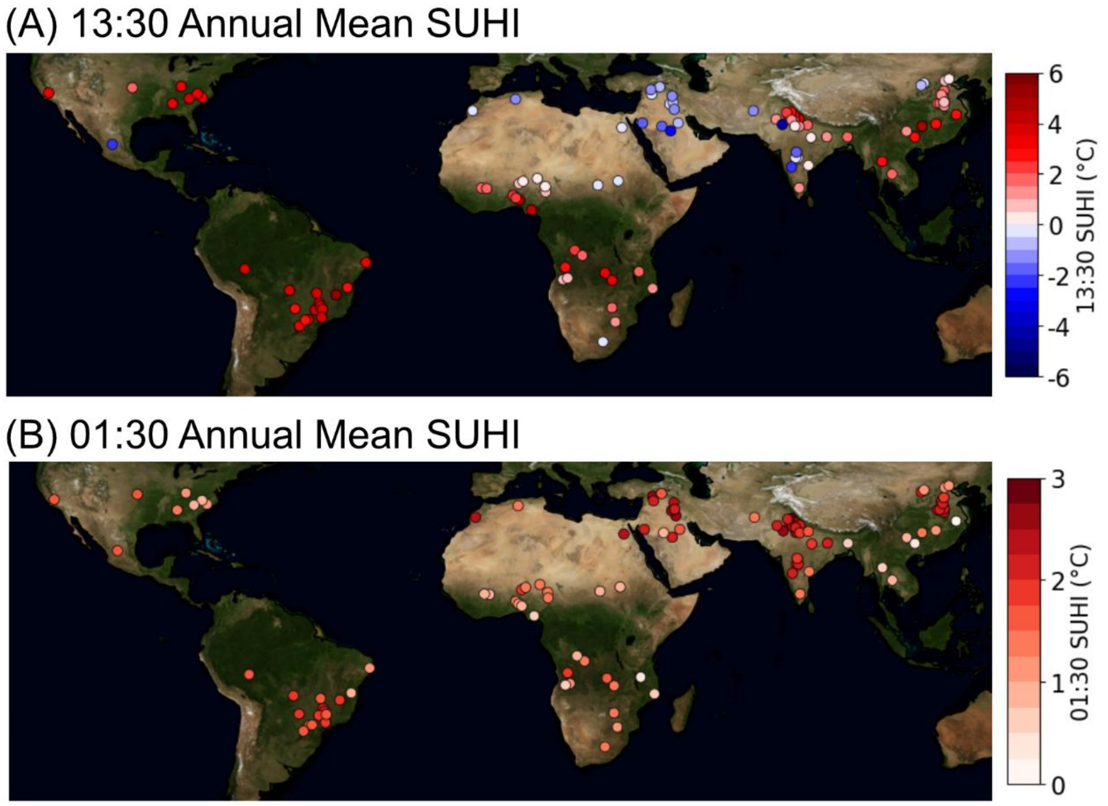

Fig. S1. Current 18-year annual mean SUHIs. (**A**) at 13:30 (daytime) and (**B**) at 01:30 (nighttime). The city annual SUHIs range from -4.0 °C to 5.4 °C for the 13:30 overpass and from 0.3 °C to 2.8 °C for the 01:30 overpass.

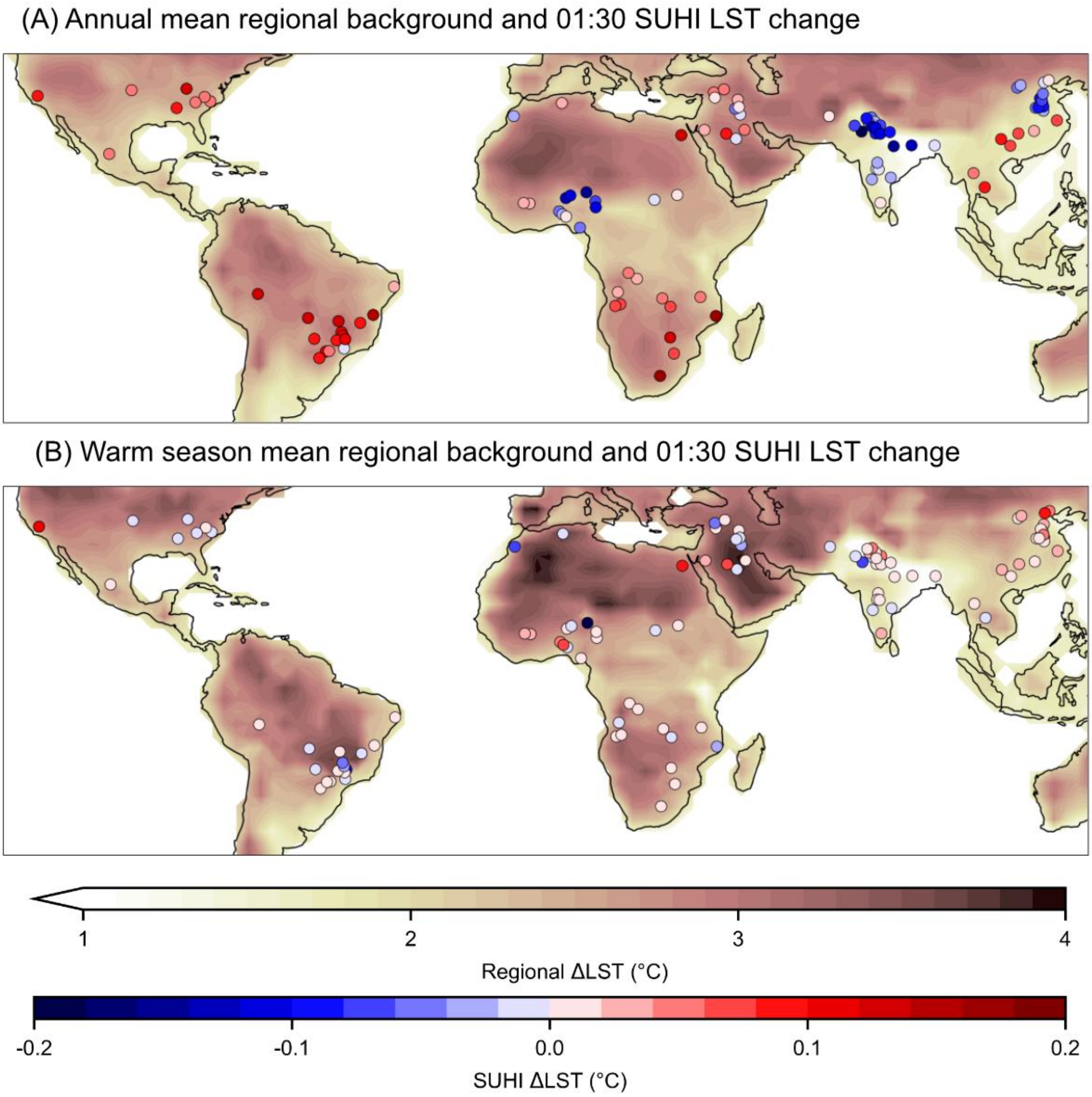

Fig. S2. A global view of the projected LST changes for the nighttime SUHI. Maps show (**A**) regional changes in annual mean LST predicted by the ESMs, with the additional 01:30 LST changes in the city. 58 % of the 01:30 SUHIs are projected to increase in their annual mean in the median ESM based changes and (**B**) regional changes in warm season mean LST predicted by the ESMs, with the additional 01:30 LST changes in the city. 63% of the 01:30 SUHIs are projected to increase in the warm season mean LST changes. There is much less city to city variation in the 01:30 SUHI, and this is reflected in the ML model test statistics of 0.65 R-squared and 0.49 °C RMSE. The RMSE is smaller than that of the 13:30 SUHI,

indicating closer predictions to the observed values. However, the R-squared, which reflects the proportion of variance explained by the model, is lower, as the model captures less variance relative to the dataset despite the improved prediction accuracy.

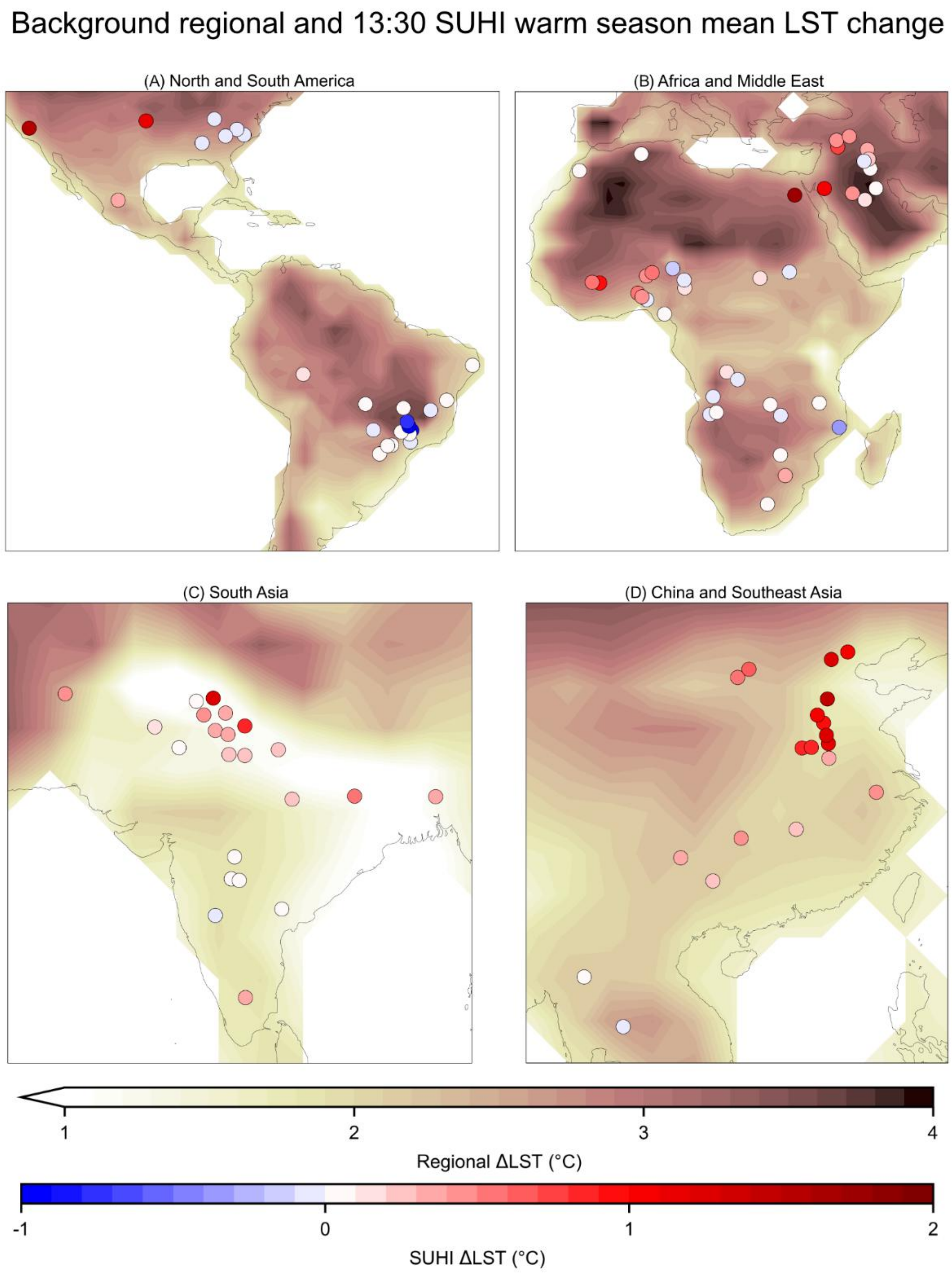

Fig. S3. A closer view of the projected warm season mean LST changes for the background regional area and the additional SUHI driven changes. The map is split into regions showing (**A**) North and South America, (**B**) Africa and Middle East, (**C**) South Asia, (**D**) China and Southeast Asia.

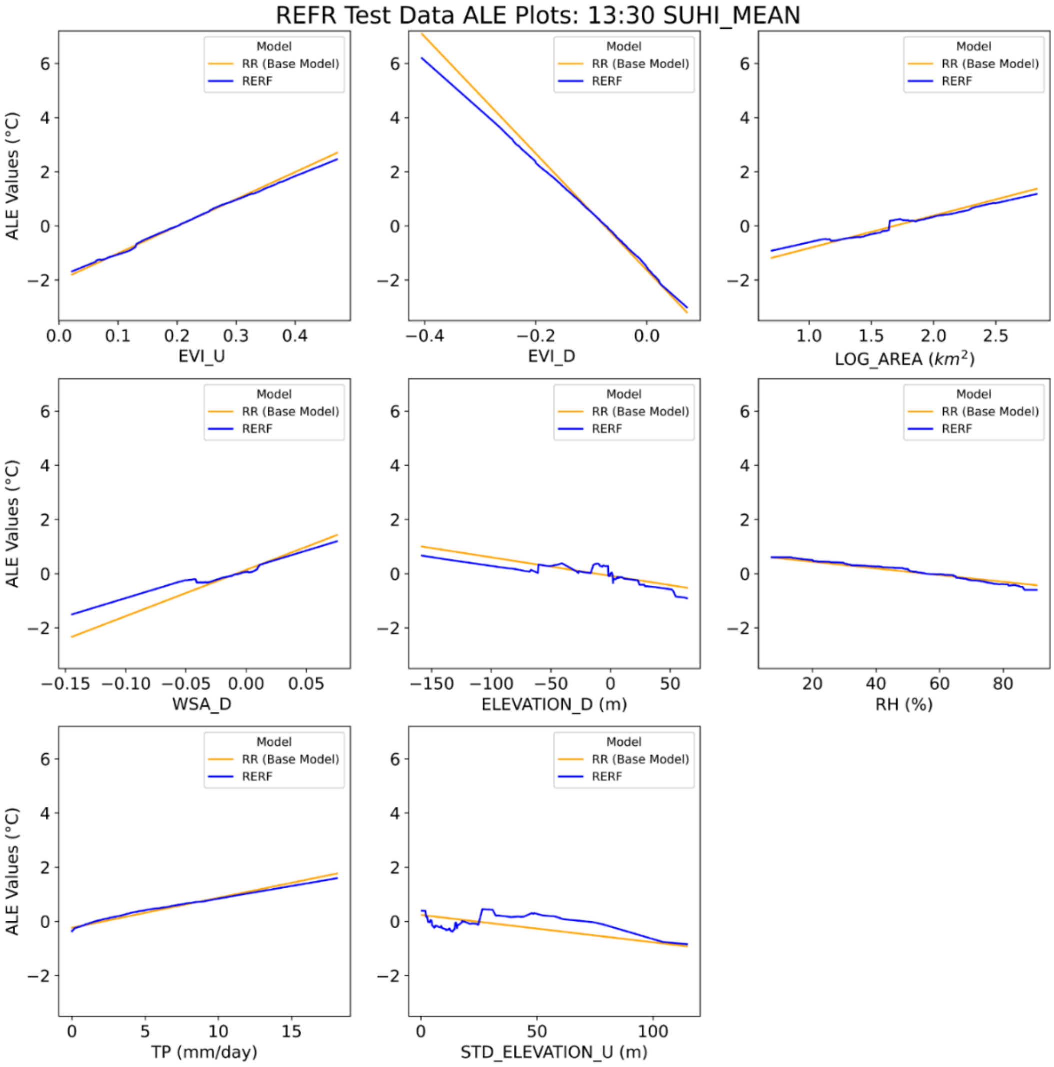

Fig. S4. 13:30 SUHI Accumulated Local Effects (ALE) plots for the REFR (blue), with the base Ridge Regression model included (in orange), showing the differences between the two models. ALE plots show how a predictor variable (on the x-axis) influences a prediction, shown on the y-axis. A positive slope means increasing the predictor variable increases the prediction and a negative slope indicates an increase in the predictor variable decreasing the prediction made. Here, changes in the EVI_D (urban minus rural EVI) variable are seen to have a strong influence on the prediction made. More rural vegetation leads to a more positive predicted SUHI, and more urban vegetation has a negative influence

on the prediction. The ALE plots were generated using test data. Training data ALE plots show the analogous relationships.

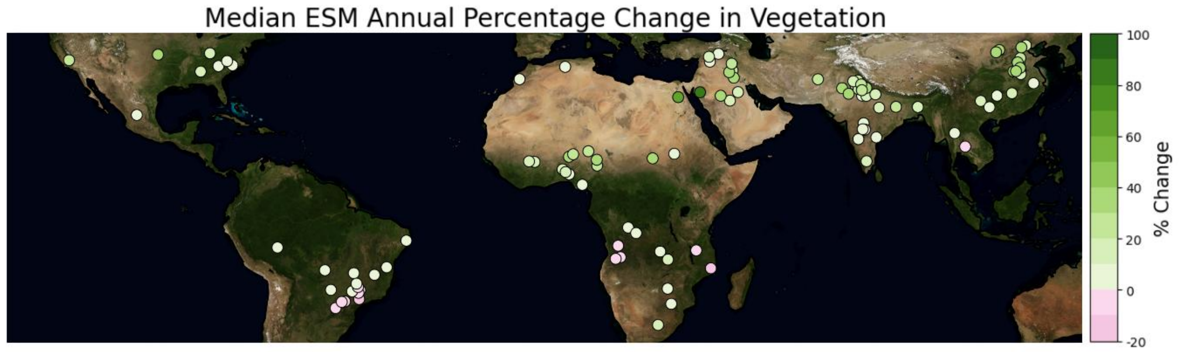

Fig. S5. The median ESM projected percentage change in vegetation. Comparison of this plot to changes in projected SUHI show areas with increasing vegetation abundance correspond to those with larger projected increases in SUHI magnitude.

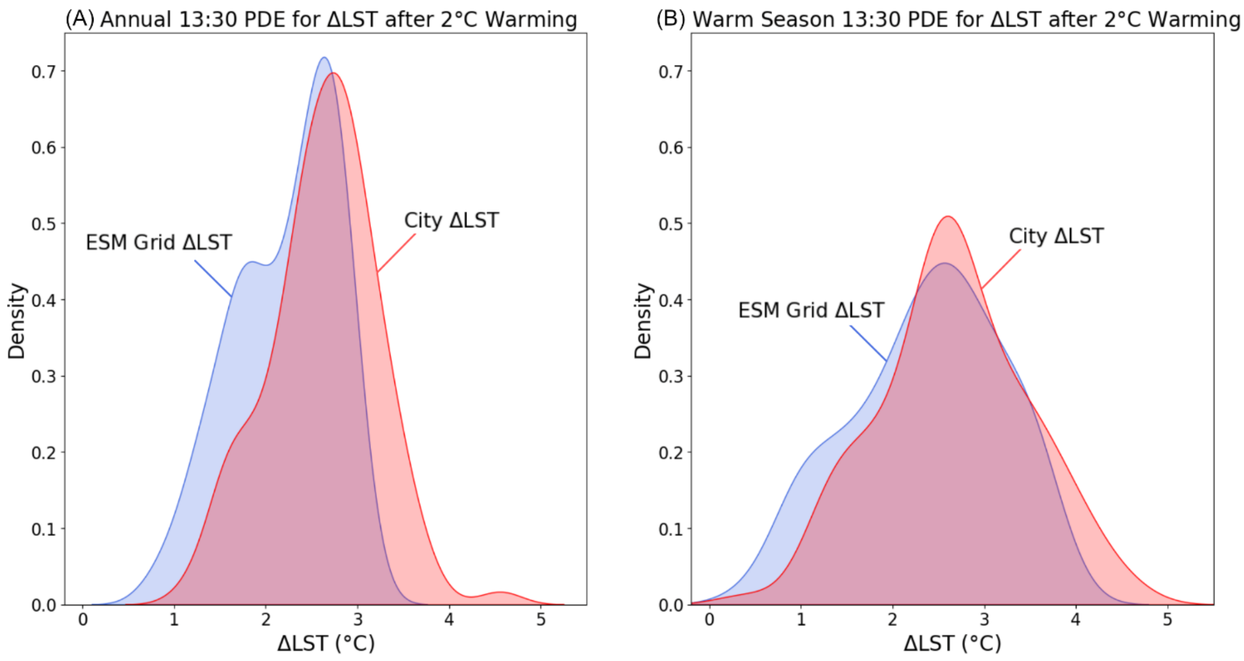

Fig. S6. Probability distributions generated by kernel density estimation to show the changes in LST for the entire region versus the changes in LST for the city. Generated for (**A**) the annual mean LST change, (**B**) the warm season LST change.

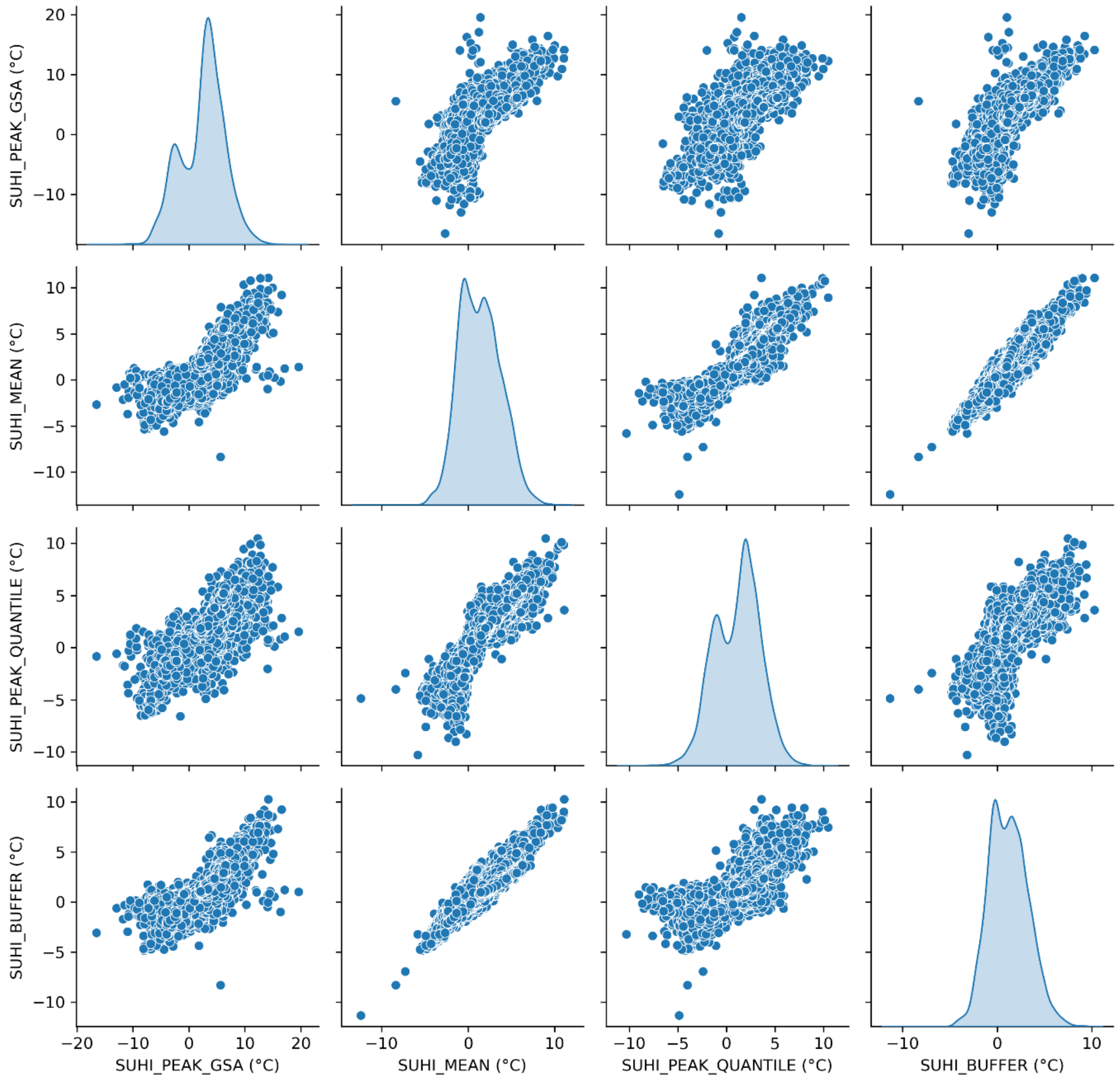

Fig. S7. Assessment of SUHI Quantification Methods. Scatterplot matrix shows correlations between the different methods of quantifying the SUHI. These include SUHI_PEAK_GSA, using the Gaussian Surface Approximation (72), SUHI_MEAN (equation 1), SUHI_QUANTILE, using the 75th percentile to determine the urban LST, and SUHI_BUFFER, which uses the same mean urban LST as SUHI_MEAN, but the rural reference is defined as 5km buffer zone from the city outline.

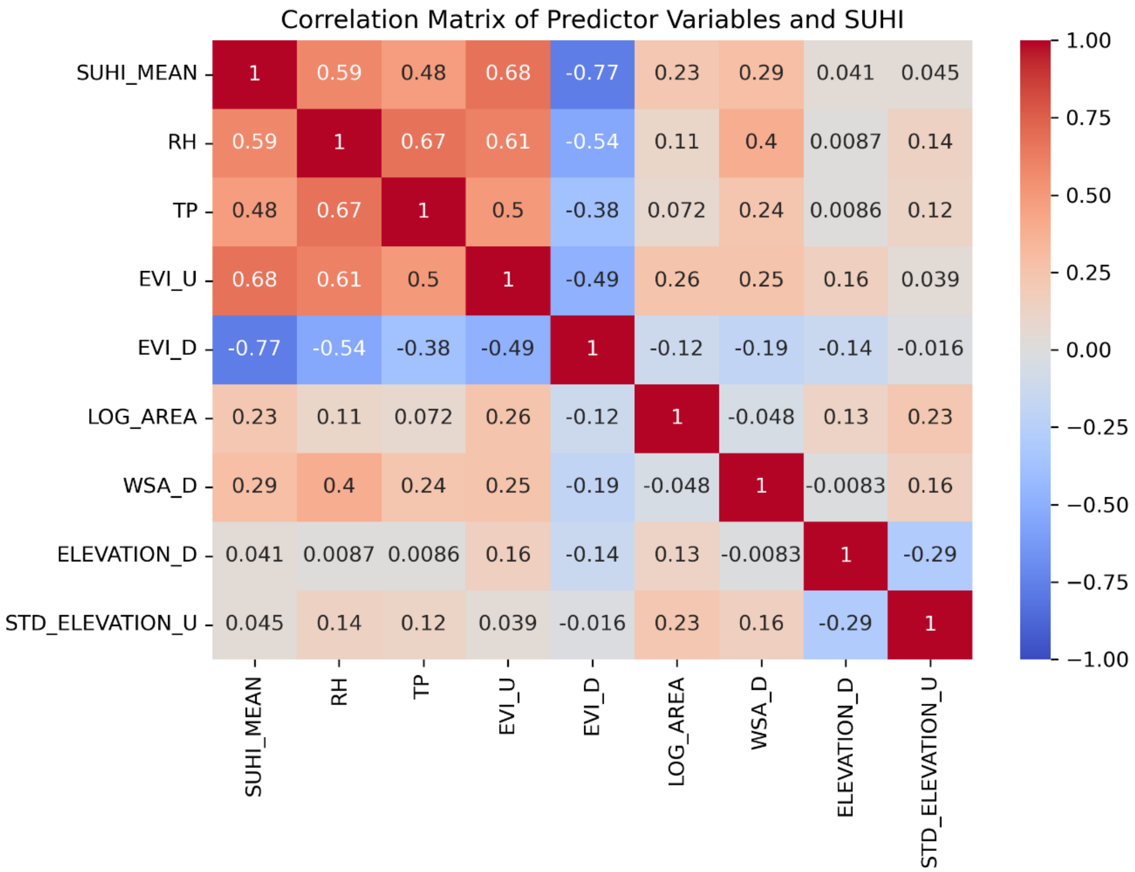

Fig. S8. Correlation Matrix between the SUHI_MEAN and predictor variables for exploratory analysis. Correlations are based on Pearson's correlations coefficients and show relationships of predictor variables with the SUHI, and across predictor variables. High positive values (red) represent a strong positive linear correlation and negative a strong negative linear correlation (blue). Light colours show a weak or negligible linear correlation (but do not discount nonlinear relationships).

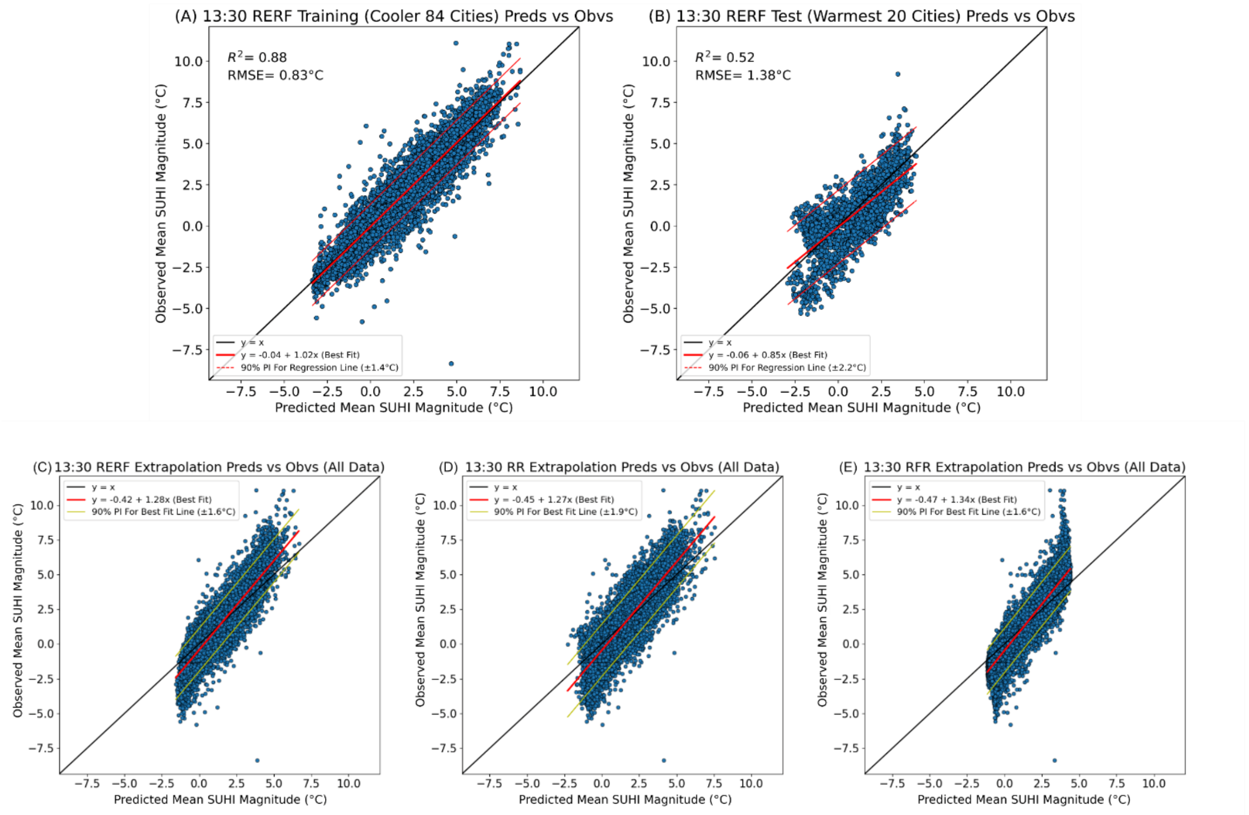

Fig. S9. Assessment of the RERFs extrapolation ability. One assessment was carried out using the warmest 20 cities (based on annual mean 2m air temperature) as the test data. The plots show scatterplots of RERF SUHI predictions (horizontal axis) versus observations (vertical axis) for (**A**) training data, the cooler 84 cities and (**B**) test data, the warmest 20 cities. Another assessment was carried out by training the middle 80% of the SUHIs based on magnitude, and testing on the most negative or smallest 10% and largest 10% combined. Performance statistics for the RERF were R-squared 0.81 (training) and 0.80 (test), and RMSE 0.73 °C (training) and 1.87 °C (test). As test data only consists of the lower and upper tails of the scatterplots, both training and test are shown on one plot, for (**C**) The RERF, and for comparison two other models, not used in this study but assessed (**D**) Ridge Regression

and (**E**) Random Forest Regression, known for its inability to extrapolate. Each point represents the monthly mean SUHI observation and prediction.
